# Inducing Acute Traumatic Coagulopathy *In Vitro*: The Effects of Activated Protein C on Healthy Human Whole Blood

**DOI:** 10.1371/journal.pone.0150930

**Published:** 2016-03-23

**Authors:** Benjamin M. Howard, Lucy Z. Kornblith, Christopher K. Cheung, Matthew E. Kutcher, Byron Y. Miyazawa, Ryan F. Vilardi, Mitchell J. Cohen

**Affiliations:** 1 Department of Surgery, University of California San Francisco and San Francisco General Hospital, San Francisco, California, United States of America; 2 Department of Surgery, University of Pittsburgh Medical Center and Presbyterian University Hospital, Pittsburgh, Pennsylvania, United States of America; Emory University/Georgia Insititute of Technology, UNITED STATES

## Abstract

**Introduction:**

Acute traumatic coagulopathy has been associated with shock and tissue injury, and may be mediated via activation of the protein C pathway. Patients with acute traumatic coagulopathy have prolonged PT and PTT, and decreased activity of factors V and VIII; they are also hypocoagulable by thromboelastometry (ROTEM) and other viscoelastic assays. To test the etiology of this phenomenon, we hypothesized that such coagulopathy could be induced *in vitro* in healthy human blood with the addition of activated protein C (aPC).

**Methods:**

Whole blood was collected from 20 healthy human subjects, and was “spiked” with increasing concentrations of purified human aPC (control, 75, 300, 2000 ng/mL). PT/PTT, factor activity assays, and ROTEM were performed on each sample. Mixed effect regression modeling was performed to assess the association of aPC concentration with PT/PTT, factor activity, and ROTEM parameters.

**Results:**

In all subjects, increasing concentrations of aPC produced ROTEM tracings consistent with traumatic coagulopathy. ROTEM EXTEM parameters differed significantly by aPC concentration, with stepwise prolongation of clotting time (CT) and clot formation time (CFT), decreased alpha angle (α), impaired early clot formation (a10 and a20), and reduced maximum clot firmness (MCF). PT and PTT were significantly prolonged at higher aPC concentrations, with corresponding significant decreases in factor V and VIII activity.

**Conclusion:**

A phenotype of acute traumatic coagulopathy can be induced in healthy blood by the *in vitro* addition of aPC alone, as evidenced by viscoelastic measures and confirmed by conventional coagulation assays and factor activity. This may lend further mechanistic insight to the etiology of coagulation abnormalities in trauma, supporting the central role of the protein C pathway. Our findings also represent a model for future investigations in the diagnosis and treatment of acute traumatic coagulopathy.

## Introduction

Trauma represents the leading cause of death in the young worldwide, and across all age groups trauma mortality surpasses that of human immunodeficiency virus, tuberculosis, and malaria combined [1]. Uncontrolled hemorrhage is the primary source of preventable early trauma mortality, and efforts to arrest and prevent such hemorrhage have become the major focus of trauma resuscitation [2–4]. Over the past decade, multiple studies have shown that at least 25% of critically injured patients present with an acute traumatic coagulopathy (ATC), which is identified at the time of admission and develops independent of iatrogenic causes [5–7]. In early investigations, this coagulopathy was associated with shock and tissue injury, and appeared to be mediated via the protein C pathway [8, 9].

Since the initial descriptions of ATC, subsequent clinical studies have produced compelling correlative data implicating the activation of protein C as a primary causal driver [10, 11]. According to this model, ATC is caused by the combination of *injury*, which leads to tissue factor exposure and subsequent thrombin production via the extrinsic coagulation pathway, and *shock*, which via hypoperfusion leads to upregulation of endothelial thrombomodulin, binding of thrombin, and activation of protein C. Once activated, protein C inactivates coagulation factors V and VIII, and disinhibits fibrinolysis by neutralizing plasminogen inhibitor 1 (PAI-1); this leads to both impaired clot formation and accelerated clot lysis [12, 13]. Patients with acute traumatic coagulopathy have prolonged PT and PTT, and decreased activity of factors V and VIII [11]; recent clinical experience indicates that they also present as hypocoagulable by viscoelastic assays such as rotational thromboelastometry (ROTEM) [14–16].

The activated protein C (aPC) hypothesis has been reproduced and tested in a mouse model of traumatic shock, with results that closely corroborate those from human clinical data [17]. Murine investigations have yielded a more precise understanding of the biochemical pathways at play, utilizing antibody blockades of specific aPC domains and histologic analysis of tissue specimens. For obvious reasons, similar controlled trials cannot be conducted in humans, and thus human data has been confined to clinical samples collected from injured trauma patients. Due in part to the difficulties of assaying aPC from human plasma samples, and to the lack of an assay for endothelial thrombomodulin activity, mechanistic assessments of ATC in humans have been limited.

To further test the etiology of traumatic coagulopathy, and to develop a potential model for future investigations, we conducted a study of the effects of activated protein C on otherwise-healthy human blood. We evaluated these effects on coagulation using standard plasma tests, factor activity levels, and ROTEM, a global viscoelastic assay of clotting dynamics. We hypothesized that the phenotype of acute traumatic coagulopathy could be induced *in vitro* in healthy human blood with the sole addition of activated protein C.

## Materials and Methods

Whole blood was drawn from 20 adult subjects, who gave verbal informed consent under a protocol approved by the University of California, San Francisco Committee on Human Research. Consent was recorded and obtained according to specifically approved parameters developed for healthy subject clinical research. Subjects were otherwise healthy, had no known prior coagulation abnormalities or diatheses, and were not taking any anticoagulant medications (including aspirin and non-steroidal anti-inflammatory agents). In order to control for any differential response based on sex, samples were collected from ten male subjects and ten female subjects. Subject age ranged from 22 to 50 years old, with mean age of 30.5 years old, consistent with the young demographic characteristic of trauma populations.

Venous blood was drawn with a 21-gauge catheter via the antecubital vein, and collected in standard glass tubes containing 3.2% buffered sodium citrate, one part citrate per nine parts blood (Tyco Healthcare Group, Mansfield MA). These tubes were maintained at room temperature for 20 minutes, then four quantities of 475 μL citrated whole blood were placed in Eppendorf tubes and mixed with increasing concentrations of purified human activated Protein C. This aPC was derived from human plasma, activated from homogenous protein C with purified alpha-thrombin (which was removed after activation), with activation confirmed by SDS-PAGE (purchased from Enzyme Research Laboratories, South Bend, IN). To ensure consistent dilution, concentrations of aPC were formulated to a constant volume of 25 μL, utilizing 20 nM Tris 0.1 M NaCl buffer. The concentrations used were based on both prior literature [18], clinical data, and our own pilot studies, as follows: control (0 ng/mL aPC), 75 ng/mL aPC, 300 ng/mL aPC, and 2000 ng/mL aPC. The mixed samples were incubated at 37°C, and ten minutes after spiking, EXTEM ROTEM was performed to assess aPC effects on the extrinsic pathway, given its central role in traumatic coagulopathy[19]. Thus each ROTEM was performed a total of 30 minutes after venipuncture, in order to standardize post-draw *in vitro* coagulation and allow for sample stabilization [20, 21]. We used the ROTEM delta machine (Pentapharm GmbH; Munich, Germany) with EXTEM reagents (Recalcifier: star-TEM®; Activator: ex-TEM tissue factor, diluted 1:200 in 10 mM Tris buffer). Each sample was warmed to 37°C in the TEM cup, prepared accordingly with the use of an automated pipette, and the test initiated within 20s of mixing of 300uL of blood with the recalcifier and activator.

The remaining whole blood from each subject was used to make four aliquots of 2850 μL, which were mixed with 150 μL of aPC at the stated concentrations, ensuring consistent dilution. After ten minutes, these aliquots were centrifuged to platelet-poor plasma; this plasma was then analyzed with a Stago Compact (Diagnostica Stago, Inc., Parsippany, NJ) to assess prothrombin time (PT), partial thromboplastin time (PTT), and coagulation factor activity profiles.

To quantify the overall amount of change in ROTEM parameters and coagulation tests by concentration, linear regression was used. Recognizing the amount of inter-subject variation in the results (including baseline/control values), and given the non-independent, subject-specific nature of the observations, a hierarchical mixed effects regression model was also employed, with clustering at the subject level. This allowed for more precise delineation of the coefficients explored in initial linear regression. For group comparisons based on concentration of aPC, Fisher’s least significant difference approach was utilized as follows: an omnibus test of the overall null hypothesis (no difference between groups) was performed for each variable using the mixed effects model; if the null was rejected, then mixed model regression coefficients specific to each group (aPC concentration) as compared to control were calculated, with resulting *p* values thus controlled for multiple comparisons. Statistical significance was set at alpha<0.05. Statistical analysis was performed by the authors using Stata Version 12.

## Results

**Fig 1:**
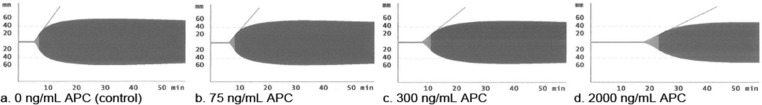
Characteristic ROTEM EXTEM tracings from a study subject. In every single one of the 20 subjects, as depicted here, increasing concentration of aPC produced ROTEM tracings consistent with worsening acute traumatic coagulopathy.

**Fig 2:**
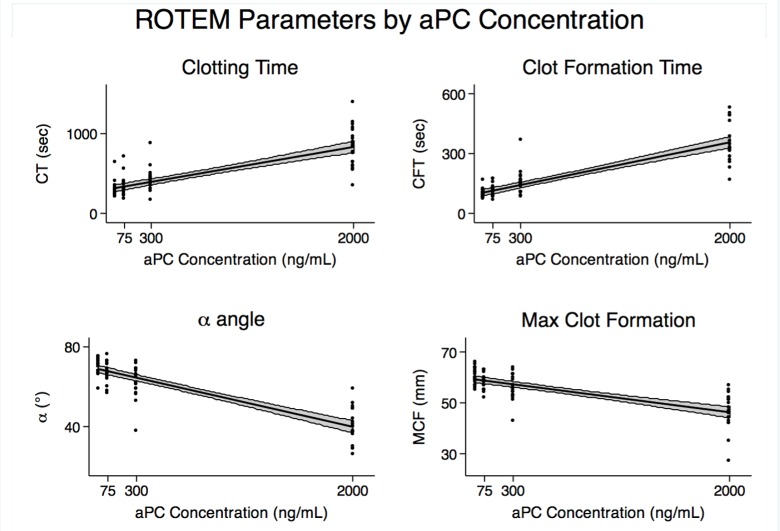
Linear regression analysis of ROTEM parameters. ROTEM EXTEM parameters changed significantly by aPC concentration, with strong linear correlation between aPC concentration and prolonged clotting time (CT) and clot formation time (CFT), decreased alpha angle (α), and reduced maximum clot firmness (MCF). The coefficients of these changes are delineated in Table 1.

In all subjects, increasing concentrations of aPC produced ROTEM tracings indicative of severely impaired coagulation (Fig 1), consistent with acute traumatic coagulopathy. ROTEM parameters differed significantly by aPC concentration, with linear prolongation of clotting time (CT) and clot formation time (CFT), decreased alpha angle (α), and reduced maximum clot firmness (MCF) (Fig 2). Mixed effects regression modeling was used to quantify these changes, in order to account for the subject-specific nature of this hierarchical data. As demonstrated in Table 1, mixed effects regression models showed that for every 100ng/mL increase in aPC concentration, clotting time, or time to clot initiation, prolonged by 26 seconds. Clot formation time, or time to reach a fixed level of clot, prolonged by 16 seconds. Alpha angle, which measures the rate of clot formation, decreased by 1.5 degrees. Early clot strength, as assayed by a10 (strength at 10 minutes) and a20 (strength at 20 minutes), was significantly reduced, by 1.11 mm and 0.84 mm, respectively. MCF, the measure of maximum clot strength, decreased by 0.65 mm. No notable inhibition of fibrinolysis was noted, with maximum lysis (ML) actually increased by 0.24% per 100 ng/mL aPC. Of note, all of these coefficients can be understood as the effect of aPC on each parameter, following correction for inter-subject variation.

**Table 1:** Mixed-effects regression modeling, change in coagulation parameters per 100ng/mL aPC.

Parameter	Coefficient*	95% CI	*p*
Clotting Time (seconds)	25.75	22.50–28.99	<0.001
Clot Formation Time (seconds)	16.17	13.10–19.24	<0.001
Alpha angle (degrees)	-1.45	-1.31–-1.60	<0.001
Clot strength at 10 min (mm)	-1.11	-0.99–1.22	<0.001
Clot strength at 20 min (mm)	-0.84	-0.74–-0.95	<0.001
Maximum Clot Formation (mm)	-0.65	-0.56–-0.73	<0.001
Maximum Lysis (%)	-0.24	-0.20–-0.29	<0.001
PT (seconds)	0.07	0.06–0.08	<0.001
PTT (seconds)	1.92	1.66–2.17	<0.001
Factor V (% activity)	-0.95	-0.81–-1.09	<0.001
Factor VIII (% activity)	-2.18	-1.73–-2.62	<0.001

* Coefficients represent change in parameter for every 100ng/mL aPC, as determined by mixed effect regression modeling, clustered by subject; mm, millimeters.

The viscoelastic findings were confirmed by conventional coagulation testing, with markedly increased prothrombin time and partial thromboplastin time at higher levels of aPC.

**Fig 3:**
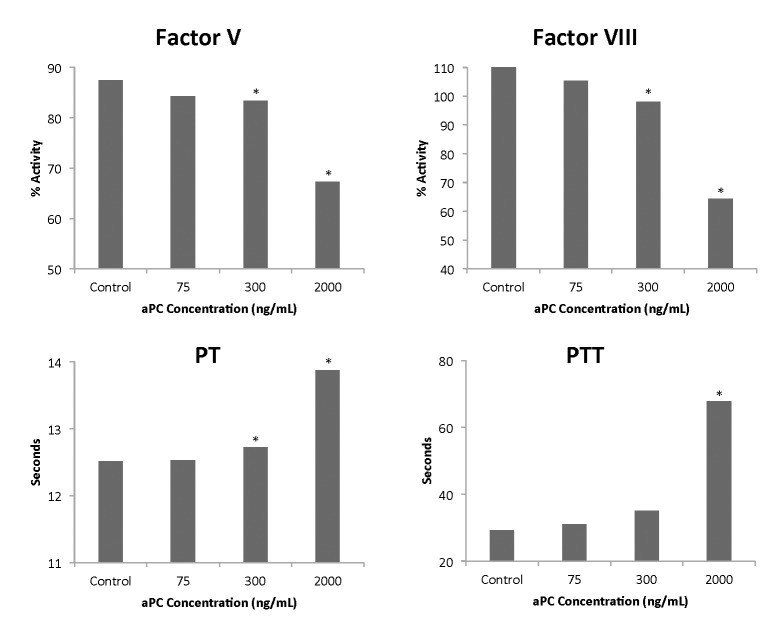
Changes in standard coagulation measures and factor activity assays by aPC concentration. Findings were confirmed in conventional plasma tests, with significantly increased PT and PTT at higher levels of APC. Corresponding decreases in Factors V and VIII are consistent with the primary anticoagulant mechanism of APC. * indicates *p*-value < 0.05 by mixed effects model by-group analysis, compared to control. PT, prothrombin time, PTT partial thromboplastin time.

The corresponding decrease in Factors V and VIII is consistent with the described primary anticoagulant mechanism of aPC. Though these relationships were significant in linear regression analysis and mixed effects regression modeling, as seen in Table 1, this was potentially driven by the extreme values found at higher concentrations of aPC; thus they are reported graphically using mixed effects by-group analysis in order to illustrate the fact that major (and statistically significant) changes occur in these test variables primarily at higher concentrations of aPC (Fig 3). Of note, no significant relationship was identified between aPC concentration and other factor activity levels, including factors II and X.

Of note, we did find differences in the effects of aPC by sex, with male blood demonstrating more change per unit aPC than female blood in several key viscoelastic parameters. This may correspond to underlying sex-based differences in hematocrit, which may lead to differing coagulation dynamics, as previously reported [22]. However, these differences were only a matter of degree, as measured by regression coefficients–the statistical significance of aPC’s effect did not change with sex. Also, when incorporated into the regression analyses as a second variable with aPC concentration, sex was not a statistically significant predictor for the major parameters of interest, and did not significantly change the magnitude or significance of other variables.

## Discussion

Since 2003, multiple trauma studies have shown that over one-quarter of severely injured trauma patients present with an endogenous coagulopathy that occurs prior to any potentially iatrogenic intervention, such as resuscitation-induced hemodilution and acidosis, or hypothermia secondary to exposure [5–7]. The emergence of the activated Protein C pathway as the driving force of this coagulopathy has been reinforced by strong correlative clinical data, including results from single and multi-center trials [11, 23]. By definition, however, clinical data are performed in real time on critically ill trauma patients, and the logistics of controlling potential confounding factors can impair the derivation of clear mechanistic findings. Even when the particular components of a biochemical pathway can be assayed in real time, they cannot be compared to pre-injury levels in patients to assess differential activation. Given the extensive cross-talk between pathways of coagulation and inflammation, analyzing specific components or processes independent of others in the post-injury milieu can be challenging, if not impossible.

With these prior limitations in mind, and seeking to expand our understanding of ATC, confirm the viscoelastic signature of aPC-mediated ATC, and establish a model for further investigation, we aimed to reproduce a phenotype of acute traumatic coagulopathy in healthy human blood by the *in vitro* addition of aPC alone. To measure the effects of aPC on the dynamics of coagulation, with special attention to the extrinsic (tissue factor) pathway, we performed ROTEM EXTEM on the spiked whole blood, with resulting tracings that were consistent with ATC at elevated aPC levels. Though the trauma community’s understanding of the specific viscoelastic manifestations of ATC remains in its nascent stages, our results conform to our clinical experience, and to both the findings of other recent clinical studies [14–16] and to murine and rat models (data submitted for publication, [24]). The clear linear correlation of increasing aPC to ROTEM parameters is consistent with the expected biological effect of aPC, and makes sense given the sensitivity of ROTEM and similar viscoelastic assays to even slight alterations in coagulation dynamics.

The suspected mechanisms of these effects were confirmed by conventional coagulation assays and measures of factor activity. The fact that such changes were not as linearly correlated with standard coagulation tests and factor assays as they were with ROTEM, and that significant changes occurred primarily at the higher concentrations of aPC, may be explained by the relative decreased sensitivity of those testing modalities to subtler changes in overall clotting mechanics. These results support the anticoagulant function of aPC via specific factor inactivation, as described in clinical studies [11, 25]. However, our findings do not correspond to the known disinhibitory effect of aPC on fibrinolysis, via inactivation of PAI-1. This may be due to the vagaries of our model (including delayed clotting initiation due to reagent dilution leads to less accurate fibrinolysis measures), though recent clinical findings have questioned the capacity of thromboelastometry to accurately detect clinical fibrinolysis at all [26]. Additionally, these effects on fibrinolysis are likely dependent upon interaction with intact endothelium, which was not a component of this model [13, 27].

In addition to its effects on the mechanisms of coagulation, activated Protein C has multiple known cytoprotective actions, which are thought to be key in reducing the pathological sequelae of uncontrolled inflammation [28, 29]. In recognition of these properties, recombinant human aPC (rhAPC) was developed as a therapeutic agent for uncontrolled sepsis, with promising initial results in a large multi-center randomized trial [30]. Though the purported benefits of such treatment were not borne out in subsequent studies, the development of rhAPC raised concerns over possible complications due to the agent’s anticoagulant properties. Nilsson and colleagues performed an rhAPC spiking study in healthy subjects to address this question, and using ROTEM found that at increasing concentrations of rhAPC, EXTEM clotting time (CT) was significantly prolonged [18]. Though they found no other changes in other ROTEM parameters, their highest concentration of rhAPC was 75 ng/mL, in an effort to replicate the plasma levels attained during therapeutic drug administration. In our study, we found that increasing concentrations of aPC had a significant effect on nearly all ROTEM parameters. Our upper concentrations were significantly higher than Nilsson’s, based on both our derived pilot concentration curves and the fact that tissue-specific levels of aPC in actively traumatized subjects remain unknown.

A recent study by Campbell and colleagues purported to definitively assess the possible effects of aPC on coagulation through a series of assays performed on blood from healthy subjects [31]. By demonstrating in specific turbidimetric and functional tests that aPC had little *in vitro* effect at levels previously reported in the clinical literature, they surmised that aPC is unlikely to be a primary causal driver of traumatic coagulopathy. However, their study was limited by its low number of subjects (total *n* of 3), a lack of endothelial component in the *in vitro* model, and most importantly, by a reliance on previously reported clinical levels of aPC. Though their experiments did suggest minimal effect from aPC at the clinically-measured picomolar level, these reported levels likely do not correspond to actual tissue concentrations in early traumatic coagulopathy. Aside from the difficulties in measuring aPC in human plasma (with no reliable standard test, and reliance upon lab-specific ELISA protocols), the exact concentration of aPC in an injured tissue bed is no doubt significantly higher than the downstream diluted concentration levels assayed via remote venipuncture several minutes (or hours) after its anticoagulant effect has been exerted. As mouse models suggest, different tissue beds may manifest coagulation, and alterations thereof, in markedly different ways [32], thus limiting the ability to draw definitive conclusions based on circulating venous blood. Given such considerations, the authors’ confidence in arriving at clinical conclusions regarding complex pathophysiology based on data from a decontextualized *in vitro* model may not be warranted.

Consistent with prior studies demonstrating differences in the manifestation of ATC based on sex [33–35], we found relative differences between the ten male and ten female subjects in this study, with increased magnitude of coefficients in male subjects, indicating more change per unit of aPC in males than females. However, aPC led to major changes in all subjects regardless of sex, and these differences of degree did not manifest as significant when sex was incorporated into regression models as a separate predictor. This effect is difficult to assess given the heterogeneity of female subjects with regard to age; data on hematocrit, menstrual stage, hormonal contraception, or menopause were not collected for this study. The potential difference in aPC-related coagulopathic changes between males and females represents an important area for future investigation.

Our study has several key limitations. First, the quantification of coagulation deficit indicated by our results should be understood in context, as our activating EXTEM reagent was diluted (based on pilot studies) and the concentrations of aPC used here may not correspond to clinical levels. As discussed above, however, aPC concentration following injury may be tissue-specific and not adequately assayed by current or previous methods. Quantitative considerations aside, the relative changes seen in our results, and the linearity of alterations in viscoelastic parameters, underscore the potent effects of aPC on clotting dynamics. Another limitation is that this *in vitro* study, by definition, lacks the endothelium found *in vivo*, which plays a major role in clinical coagulation and inflammation. Thus subsequent steps will include integrating the model with an endothelial element. However, the ability to explore the effects of aPC independent of endothelial elements may be useful as well, as when assessing the effects of antibody-targeted blocking of specific functional enzymatic domains. Lastly, the mechanisms of coagulopathy following trauma are extraordinarily complex and likely involve the interplay of multiple biochemical pathways; this model should thus be seen as a tool for exploring one major mechanistic hypothesis, and not as definitive or wholly representative of the pathophysiology of ATC.

In sum, we successfully reproduced a phenotype resembling acute traumatic coagulopathy in healthy human blood by the *in vitro* addition of aPC alone, as evidenced by viscoelastic measures and confirmed by conventional coagulation and factor activity assays. These findings lend further mechanistic insight to the etiology of coagulation abnormalities in trauma, supporting the central role of the protein C pathway. Our findings also represent a model for future investigations in the diagnosis and treatment of acute traumatic coagulopathy.
